# College and Hospital Notes

**Published:** 1910-01

**Authors:** 


### COLLEGE AND HOSPITAL NOTES.

THE Buffalo General Hospital staff was reorganised in part at a meeting of the trustees held at noon December 23, 1909. The office of surgeon-in-chief was created. President Pardee announced bequests of $2,000 from Susan E. Kimberly and $1,000 from J. Ambrose Butler. The staff as now organised follows:

Dr. Roswell Park, surgeon-in-chief; Dr. Marshall Clinton and Dr. Edgar R. McGuire, attending surgeons; Dr. Frederick Parmenter, assistant attending surgeon; Dr. Nelson W. Wilson and Dr. Thomas B. Carpenter, genitourinary surgeons; Dr. W. W. Plummer, orthopedic surgeon; Dr. Marcell Hartwig, Dr. William Warren Potter, Dr. John Parmenter, Dr. William C. Phelps, consulting surgeons; Dr. Herbert A. Smith, assistant attending gynecologist.

Dr. Henry R. Hopkins, who resigned from the active staff was appointed a consulting physician and Dr. Rochester was appointed to take Dr. Hopkins's place. At the next meeting of the trustees the names of the medical staff will be announced.

A resolution in memory of Edwin Townsend Evans was adadopted.

